# Joint visualization of seasonal influenza serology and phylogeny to inform vaccine composition

**DOI:** 10.3389/fbinf.2023.1069487

**Published:** 2023-03-22

**Authors:** Jover Lee, James Hadfield, Allison Black, Thomas R. Sibley, Richard A. Neher, Trevor Bedford, John Huddleston

**Affiliations:** ^1^ Vaccine and Infectious Disease Division, Fred Hutchinson Cancer Center, Seattle, WA, United States; ^2^ Chan Zuckerberg Initiative, San Francisco, CA, United States; ^3^ Biozentrum, Universität Basel, Basel, Switzerland; ^4^ Swiss Institute of Bioinformatics, Lausanne, Switzerland; ^5^ Howard Hughes Medical Institute, Seattle, WA, United States

**Keywords:** influenza, serology, phylogeny, interactive, visualization, pandemic

## Abstract

Seasonal influenza vaccines must be updated regularly to account for mutations that allow influenza viruses to escape our existing immunity. A successful vaccine should represent the genetic diversity of recently circulating viruses and induce antibodies that effectively prevent infection by those recent viruses. Thus, linking the genetic composition of circulating viruses and the serological experimental results measuring antibody efficacy is crucial to the vaccine design decision. Historically, genetic and serological data have been presented separately in the form of static visualizations of phylogenetic trees and tabular serological results to identify vaccine candidates. To simplify this decision-making process, we have created an interactive tool for visualizing serological data that has been integrated into Nextstrain’s real-time phylogenetic visualization framework, Auspice. We show how the combined interactive visualizations may be used by decision makers to explore the relationships between complex data sets for both prospective vaccine virus selection and retrospectively exploring the performance of vaccine viruses.

## 1 Introduction

Seasonal influenza A/H3N2 viruses primarily evolve by acquiring mutations that allow them to escape antibodies from previous infections or vaccinations (Petrova and Russell, 2018). This process, known as antigenic drift, changes the appearance of viral surface proteins hemagglutinin and neuraminidase. Hemagglutinin (HA) is the primary target of our adaptive immunity and the primary component of the seasonal influenza vaccine. Therefore, continued antigenic drift in HA necessitates regular updates to the seasonal influenza vaccine.

The World Health Organization (WHO) Global Influenza Surveillance and Response System (GISRS) tracks antigenic drift throughout the year by sequencing the HA gene of circulating viruses, growing candidate vaccine viruses in cell lines and chicken eggs, and performing serological experiments (Morris et al., 2017). HA gene sequences reveal “clades” or groups of recent influenza viruses that descend from a common ancestor. When the common ancestor of a clade carries amino acid mutations at positions in HA that have been previously targeted by antibodies following infection or vaccination (Wolf et al., 2006; Shih et al., 2007; Koel et al., 2013), viruses in that clade may be able to escape our existing immunity. GISRS researchers select representative viruses from major clades to grow in the cell line and chicken egg environments used in vaccine production (Katz et al., 2011). Viruses that grow in these conditions become candidate vaccine viruses or vaccine candidates. Serological experiments measure antigenic drift by quantifying how well viruses from one clade can escape detection by antibodies against vaccine candidates from the same clade or other clades. These experimental measurements validate the effects of specific mutations on antigenic drift.

The gold standard of these serological experiments is the hemagglutination inhibition (HI) assay (Hirst, 1943). HI assays measure the minimum concentration or “titer” of antibodies required to prevent a given test virus from binding to and, thereby, agglutinating red blood cells. Typically, antibodies come from previously uninfected ferrets who are then infected with a specific reference virus (e.g., a vaccine candidate). Each HI assay requires a series of two-fold dilutions of ferret antibodies to determine the minimum titer that inhibits agglutination. The higher the antibody titer required to prevent agglutination, the more antigenically drifted the test virus is from the reference virus. To enable comparison between measurements from different reference viruses, researchers normalize titers by the titer required to inhibit the reference virus itself and convert values to a log_2_ scale (Neher et al., 2016). The resulting values represent the magnitude of antigenic distance between a given reference virus and other test viruses. Traditionally, viruses with an antigenic distance greater than 2 log_2_ units are considered antigenically distinct (Katz et al., 2011).

The WHO convenes influenza vaccine composition meetings (VCMs) twice per year with each meeting occurring approximately 9 months prior to the next influenza season in a given hemisphere (Morris et al., 2017). At these meetings, WHO decision makers must select a single vaccine virus for H3N2 from the pool of available vaccine candidates. This selection strongly depends on the patterns of antigenic drift present in HI titers for clades identified from HA gene sequences. Key questions that decision makers must answer in this process include a) which available vaccine candidates require additional titer measurements against currently circulating clades, b) which vaccine candidates have the lowest antigenic distance to each clade, and c) what is the antigenic diversity of recent clades? The best vaccine candidate will have titer measurements against all current clades and have the lowest antigenic distance across these clades. Such candidates are said to effectively “cover” currently circulating clades. Determining the vaccine candidate that most effectively covers recent clades requires a direct comparison of antigenic distances across all available vaccine candidates.

**FIGURE 1:**
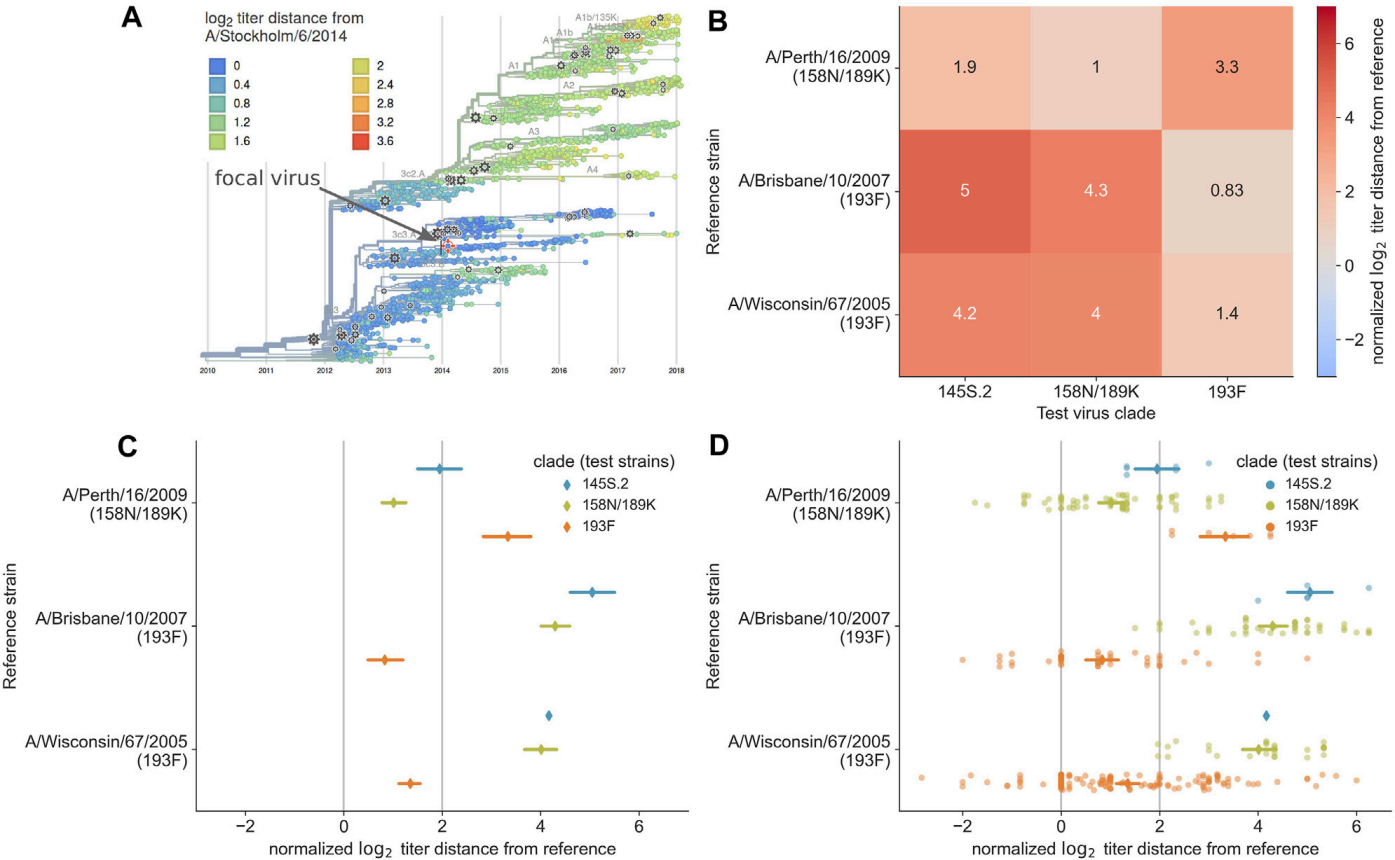
Previous approaches to static visualization of serological data for seasonal influenza vaccine composition reports. **(A)** Phylogenetic visualization (Neher and Bedford, 2018) allows the user to select a single vaccine candidate (e.g., A/Stockholm/6/2014) and see how well that virus might cover other circulating viruses in their genetic context based on the antigenic distance encoded by color (orange and red color indicate greater distance and less coverage by the selected virus). To compare multiple vaccine candidates, users have to select different reference viruses manually and toggle between them. **(B)** Heatmap visualization of mean antigenic distances between multiple vaccine candidates (reference viruses on the *y*-axis) and viruses in currently circulating phylogenetic clades. Heatmaps encode distance by color and text, allowing the user to compare how well multiple vaccine candidates might cover circulating viruses. **(C)** Interval plot of mean ±89% confidence interval values of antigenic distances between vaccine candidates (*y*-axis) and viruses in currently circulating clades. Unlike the heatmap visualization, the interval plot encodes distance with a positional encoding (the *x*-axis) instead of color and encodes clades with color. The vertical gray lines represent the threshold above which viruses are considered antigenically distinct (line at x = 2) and where viruses are antigenically identical (line at x = 0). This view allows users to compare multiple vaccine candidates, identify the candidate that covers specific clades based on a mean value to the left of the threshold at x = 2, and view the variance in the underlying HI measurements. **(D)** Combined swarm and interval plot showing the raw pairwise measurements between each vaccine candidate and the test viruses in each clade. This view allows users to perform the same tasks as the interval plot, but it also allows users to identify how many measurements support the summary statistics for a given vaccine candidate and identify multiple modes in the raw data distribution that could indicate within-clade antigenic variation.

Historically, WHO decision makers have answered the key questions above using standard phylogenetic visualizations from HA gene sequences (Felsenstein, 2003; Lemey et al., 2012) and separate visualizations of antigenic evolution from HI assays (Smith et al., 2004). Visualization of antigenic evolution in a phylogenetic context is a relatively recent development (Steinbrück and McHardy, 2012; Bedford et al., 2014; Neher et al., 2016). We previously developed two alternate visualizations of antigenic data to inform vaccine selection through reports to the WHO (Bedford and Neher, 2018; Bedford et al., 2019). The first visualization is an interactive phylogenetic view implemented in the genomic epidemiology tool *nextflu* (Neher and Bedford, 2015; 2018). When the user selects a given reference virus from the phylogeny (by selecting a “gear” icon), the tool plots the pairwise antigenic distances between that reference and the corresponding test viruses in the tree using color to represent the distance values (Figure 1A). This representation reveals clades that the selected reference virus may or may not cover and which clades lack measurements against the reference. While this view provides phylogenetic context of titer measurements and shows the individual pairwise measurements, it does not support direct comparison of antigenic distances from different vaccine candidates. Instead, users must quickly toggle between different vaccine candidates in the tree to get a sense of how well different viruses may perform.

To complement the interactive phylogenetic visualization, we also developed a static heatmap visualization that summarizes the mean antigenic distances between a subset of vaccine candidates and all test viruses within each extant clade. The resulting heatmaps use the *x*-axis to encode the names of major clades, the *y*-axis to encode the names of reference viruses, and color and text to encode the mean log_2_ distance between a given reference virus and corresponding test viruses in each clade (Figure 1B). These heatmaps allow decision makers to directly compare average antigenic distances for different vaccine candidates and quantify how well these candidates cover extant clades. However, these heatmaps suffer reduced expressiveness by encoding the most valuable quantitative data with color instead of a positional encoding. Additionally, this view shows a summary statistic instead of the underlying distributions of the data, concealing the number and variance of measurements for each reference virus.

To overcome the limitations of existing antigenic visualizations, we applied user-driven design based on the goals of decision makers described above and used standard visual design principles to produce a more expressive and effective visualization for influenza virologists. The result is a new component of Nextstrain’s interactive phylogenetic visualization platform that we call the measurements panel. Below, we describe the measurements panel and provide two case studies that demonstrate the practical value of this tool for vaccine composition decisions with the interactive visualizations available at nextstrain.org/community/blab/measurements-panel/flu/seasonal/h3n2/ha.

## 2 Methods

### 2.1 Visual design

Between the interactive phylogenetic view in *nextflu* (Figure 1A) and the static heatmap visualization used in WHO reports (Figure 1B), the heatmaps addressed the most user goals. The only original goal that heatmaps could not address was the communication of the number of measurements available for each clade that support the summary statistic of mean titer distance. Additionally, both of these prior visualizations either obscured or hid the underlying distributions of the raw data. Since visualization of these distributions can improve confidence during the decision-making process (Correll and Gleicher, 2014; Hullman et al., 2015; Fernandes et al., 2018), we treated the need to view these distributions as an auxiliary user goal.

In the context of visual design principles, both the phylogenetic and heatmap views encode the most relevant quantitative data of antigenic distance with color. However, quantitative data can be more effectively represented by positional encodings (e.g., *x*- or *y*-axis positions) whereas nominal data (e.g., names of phylogenetic clades) can be effectively encoded with color (Mackinlay, 1986). In *nextflu*’s phylogenetic view, the two available positional axes represent time and the unitless phylogenetic position of nodes, neither of which are relevant to the user goals described above. In the heatmaps, the two positional axes encode two nominal data types.

We reasoned that we could make a more effective visualization that addressed all user goals by simply changing the encoding of data in the heatmaps. To this end, we swapped the encoding of antigenic distances and test clades, encoding quantitative distances on the *x*-axis positional scale and encoding nominal test clades with a color scale. The positional encoding of antigenic distances allowed us to visually encode relevant thresholds for decision-making (e.g., *x* = 2 log_2_), show all available measurements for each reference virus at once, and display a summary statistic (mean and standard deviation of antigenic distances) for each reference virus. We retained the encoding of nominal reference virus names on the *y*-axis, since most user goals require comparison of distances between specific vaccine candidates.

### 2.2 Implementation

We implemented this design as a new interactive measurements panel within Nextstrain’s visualization tool, Auspice (version 2.43.0), which is freely available on GitHub (github.com/nextstrain/auspice) under the AGPLv3 license. Auspice is a phylogenetic visualization platform inspired by *nextflu* and which maintains the interactive data exploration, with the measurements panel appearing alongside and in-sync with other views into the data (currently phylogenetic, geographic and genomic diversity views). The visualization requires a minimum of two JavaScript Object Notation (JSON) files that are produced by the Nextstrain bioinformatics toolkit, Augur (Huddleston et al., 2021). The phylogenetic tree is provided *via* a dataset JSON file produced by augur export v2 and the measurements data is provided *via* a measurements sidecar JSON file produced by augur measurements. The measurements file must follow a specific filename format for Auspice to link it to the dataset file, where the dataset filename is ${name}.json and the measurements filename must be ${name}_measurements.json. The measurements sidecar JSON is expected to have an array of collections, where each collection contains its display configurations for Auspice and an array of measurements. Each measurement must include a numeric value to plot along the *x*-axis and a strain name that exactly matches a sequence name in the phylogenetic tree to support interactivity between panels. For complete technical details about the data structure used by the measurements panel, see the measurements sidecar JSON schema document. The application can be cloned and run locally or users can use Auspice through two public websites. Users can drag and drop dataset and measurements files onto auspice.us/ to visualize the data locally in their own browser. Visualizations can be also shared with others through nextstrain.org/. Full documentation for sharing analyses through Nextstrain can be found at docs.nextstrain.org/page/guides/share/.

### 2.3 Data curation and analysis

We evaluated the new measurements panel by constructing a Nextstrain analysis (Hadfield et al., 2018) with previously published HA gene sequences and HI titers (Bedford et al., 2014). A full data curation guide is available online at github.com/blab/measurements-panel/tree/main/data#readme and a full guide to running the bioinformatics analyses is available at github.com/blab/measurements-panel#readme. Briefly, we downloaded HI titer data and accessions for H3N2 HA sequences from Bedford et al. (2014)’s GitHub repository. We downloaded and combined HA sequences from the Influenza Virus Resource or GISAID, depending on the original source. We parsed metadata including viral sample name, database accession, collection date, and sequence authors from the sequence headers with augur parse. We aligned HA sequences with mafft v7.508 (Katoh and Standley, 2013), inferred a phylogenetic tree with IQ-TREE 2.2.0.3 (Minh et al., 2020), and inferred a time tree with TreeTime 0.9.4 (Sagulenko et al., 2018). We annotated mutations on the phylogenetic tree and constructed the measurements panel data JSON with Augur 21.0.0 (Huddleston et al., 2021). For improved reproducibility, we automated the execution of these tasks in a Snakemake workflow (Mölder et al., 2021).

In the absence of official WHO clade designations for the time period of this analysis, we algorithmically assigned realistic clade labels to each internal node of the HA phylogeny that a) carried a mutation at one or more of seven previously identified sites associated with antigenic drift (Koel et al., 2013) and b) circulated at or above 10% global frequency at some point during its existence. This algorithm mimics the decision-making approach used to assign clade labels in the last decade based on genetic data and prior knowledge about potentially relevant antigenic sites.

To construct the measurements panel JSON, we first normalized the raw HI titer measurements with Augur’s implementation of the titer substitution model (Neher et al., 2016). Normalization log_2_-transforms raw titer measurements and subtracts the transformed measurement for test virus *a* and reference serum *β* raised against reference virus *b* from the corresponding measurement for the reference virus *b* and its serum *β*. This normalization produces an antigenic distance between test and reference viruses that we can compare across HI experiments. Next, we converted these distances to a tab-separated values (TSV) file with a custom Python script (scripts/get_antigenic_distances_between_strains.py) and ran the new augur measurements export command with this TSV file as input. Using a collection configuration file passed to the augur measurements export command, we sorted grouping labels in the measurements JSON file for reference strain, reference clade, and serum by descending order of each clade’s minimum *y*-axis position in the phylogeny and then by ascending alphabetical order of reference strain name within each clade. This sorting causes reference strains and other grouping labels to appear in the measurements panel in the same order each reference clade first appears in the phylogeny, keeping closely related clades adjacent to each other in the panel. We sorted the grouping labels for each measurement’s “source” by the default which is in descending order by the number of measurements in the grouping. The resulting visualization for this paper has been shared *via* Nextstrain community and can be viewed at nextstrain.org/community/blab/measurements-panel/flu/seasonal/h3n2/ha.

## 3 Results

### 3.1 Interactive visualization of titer measurements

**FIGURE 2:**
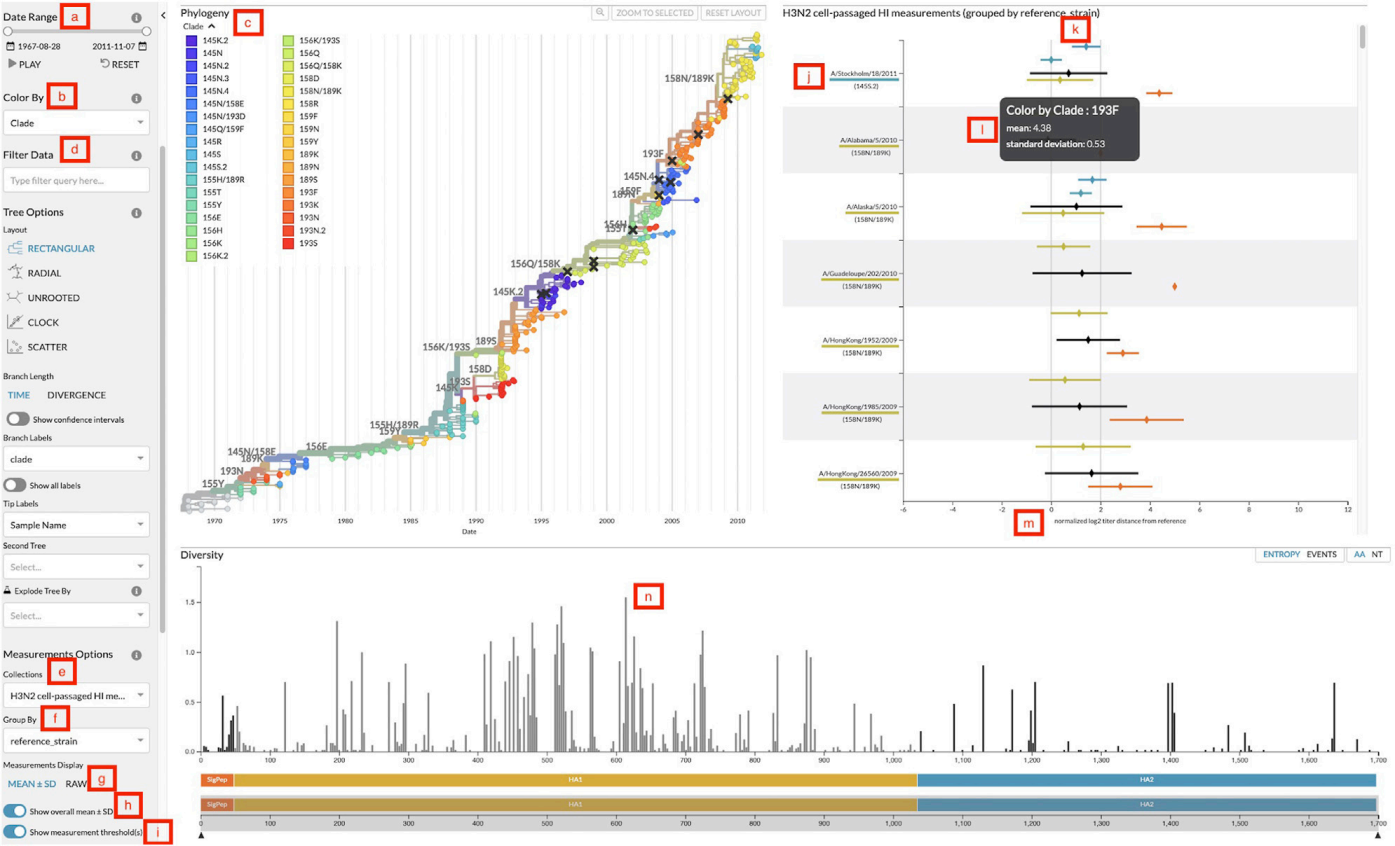
Screenshot of Auspice with the sidebar controls, the phylogenetic tree panel, the measurements panel, and the diversity panel. **(A)** Date range slider to filter both tree and measurements by the test virus sample date. **(B)** Color-by dropdown to change the attribute to use for coloring the tree and measurements. **(C)** Legend for colors and their corresponding values linked to viruses in the tree and measurements. **(D)** Data filter search bar to filter tree and measurements data by specific attributes. **(E)** Measurements collection dropdown to change the collection of measurements displayed. **(F)** Measurements group by dropdown to change the grouping of measurements data. **(G)** Toggle to change measurements display between mean with standard deviation and individual raw measurements. **(H)** Toggle to display or hide the overall mean and standard deviation for each group. **(I)** Toggle to display or hide the threshold lines. **(J)** Grouping label for each group of measurements. Labels appear in descending order by the number of measurements in that group (default), by the custom order defined in a configuration file passed to augur measurements export, or in the order they have been selected by a user filter. This particular view uses reference viruses that are also present in the tree so the label includes the virus’s corresponding color and color-by value. **(K)** The threshold lines for titer measurements to indicate two thresholds: antigenically identical viruses at x = 0 and antigenically distinct viruses at x = 2. **(L)** The tooltip that displays more details for the hovered measurement. This particular view hovers over a mean and standard deviation. **(M)** The *x*-axis of the measurements plot shows the range of measurement values in this collection. **(N)** Clicking on a bar in the diversity panel updates the tree and measurements to color by genotypes at that position.

Our goal for incorporating the measurements panel into Auspice is to allow users to explore relationships between the genetic and serological data in one interactive visualization. Test viruses of the titer data are directly linked by name to the viruses displayed in the phylogenetic tree, to ensure that any interactions with the tree also affect the measurements displayed. When users filter the tree by date (Figure 2A), metadata attributes of viruses (Figure 2D), or subtrees selected by clicking on a corresponding tree branch, the measurements panel updates to reflect only the matching test viruses. Users can easily focus on a subset of phylogenetically relevant measurements and examine the measurements of test viruses in recently circulating clades. The measurements are colored by the same coloring attribute (Figure 2B) as the phylogenetic tree, adding the legend values (Figure 2C) as another dimension of nominal data to the titer measurements. This coloring is especially useful for viewing titer data by genotypes of test viruses, allowing users to inspect relationships between specific mutations in HA and antigenic drift quantified by titer measurements. The investigation of genotypes is further facilitated by the diversity panel in Auspice, which shows the diversity of alleles across the genome. Clicking on a bar in the diversity panel will change the coloring of the tree and measurements to the genotypes at that position (Figure 2N).

The measurements JSON can include multiple collections of titers for a single phylogenetic tree and users can change the collection displayed with the collection dropdown (Figure 2E). Users can then review different sets of data for the same phylogenetic tree such as separate measurements of cell- and egg-passaged virus titers. Within each collection, users can compare measurements across different groupings by changing the grouping category with the “group by” dropdown (Figure 2F). For vaccine selection, grouping by the reference virus (Figure 2J) allows decision makers to directly compare titers across multiple vaccine candidates. Other groupings such as data source or ferret serum can be used to explore the variability of the titer measurements. By default, groupings appear in descending order by the number of measurements in each group. Users can specify a custom order for grouping values with a configuration file passed to augur measurements export. Users can also manually assign an order for specific groupings in Auspice by filtering measurements to the corresponding grouping values (Figure 2D). Groupings will appear on the *y*-axis in the order that the user selects them from the filter field. The overall mean for each grouping can then be toggled (Figure 2H) for whole group comparisons.

The data display can be switched between mean with standard deviation and raw individual measurements (Figure 2G). The means are calculated per color attribute to allow for comparisons across attributes within each group. This view maintains the ability to view the mean antigenic distance for test viruses within each clade that we had implemented in the static heatmaps. The raw measurements view plots each individual measurement to give users a detailed view of the quantity and distribution of titers, which can inform design decisions for future titer experiments. The titers thresholds can be toggled (Figure 2I) to add a clear demarcation of the threshold value for a view of when titer measurements are considered antigenically identical and distinct (Figure 2K). We discuss the application of these features in detail in two case studies in the following sections.

### 3.2 Case study 1: Retrospective vaccine selection in fall 2009

As noted above, the WHO convenes VCMs twice per year (Morris et al., 2017). The northern hemisphere VCM occurs in February or March ahead of a winter season in October through April. The southern hemisphere VCM occurs in September or October ahead of a winter season in April through October. To demonstrate the utility of an interactive visualization of serological measurements for vaccine composition decisions, we performed a retrospective analysis of a H3N2 vaccine update made for the southern hemisphere in the fall of 2009. We used publicly available sequence and titer data (Bedford et al., 2014) to reconstruct a H3N2 HA phylogeny and measurements panel representing information that was available at the time of the VCM (see Methods). We note that the actual selection process used a richer dataset which is not publicly available to include here, and as such these data should be seen as representative of the process only.

**FIGURE 3:**
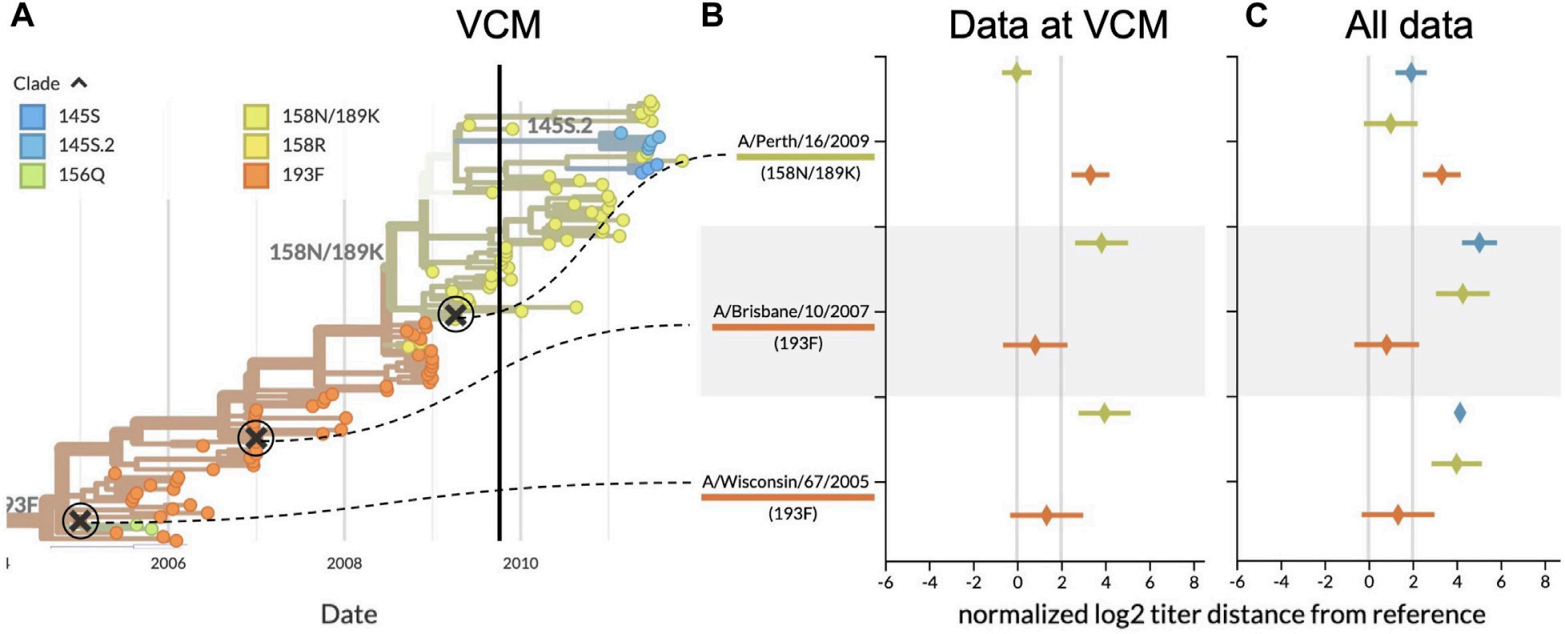
Serological analysis of H3N2 clades around the time of the southern hemisphere vaccine composition meeting (VCM) for the 2010 influenza season. **(A)** Phylogenetic tree summarizing the clades circulating before and after the VCM in September 2009. At the time of the VCM, clade 158N/189K (yellowish green) was dominant, and A/Perth/16/2009 was the vaccine candidate from this clade. A/Brisbane/10/2007 (clade 193F) had been the southern hemisphere vaccine in 2008 and 2009 and A/Wisconsin/67/2005 (clade 193F) had been the vaccine in 2007. **(B)** HI titers for three vaccine candidates against test viruses from the two largest recent clades, using only data available prior to the final decision from the VCM (25 September 2009). HI titers greater than 2 log_2_ indicate the inability of previous vaccines to cover viruses from the most recent clades. **(C)** Same as **(B)** but including test viruses and HI measurements collected after the VCM for 158N/189K and the largest new clade with HA1:145S, 145S.2. With mean HI titers less than 2 log_2_, A/Perth/16/2009 covered 158N/189K viruses better than the previous vaccines.

The VCM ahead of the 2010 southern hemisphere season occurred in September 2009. One major clade circulated at that time, 158N/189K, that descended from the previously dominant clade 193F (Figure 3A). The most recent H3N2 vaccine virus, A/Brisbane/10/2007, had been used for the two prior influenza seasons (2008 and 2009) and represented the older 193F clade (WHO, 2010). Prior to the A/Brisbane/10/2007 vaccine, A/Wisconsin/67/2005 had been the vaccine virus in the 2007 southern hemisphere season. While 193F dominated the 2008–2009 season, 158N/189K appeared to be dominant in September 2009. At the time of the VCM, titers for both A/Brisbane/10/2007 and A/Wisconsin/67/2005 against test viruses from 158N/189K exceeded the 2 log_2_ threshold (Figure 3B), indicating their inability to cover this recent clade. In contrast, a newer vaccine candidate from the 158N/189K clade, A/Perth/16/2009, had a titer distance of −0.01 ± 0.67 log_2_ units (mean ± standard deviation) against test viruses from the same clade. These results strongly supported an update from the previous vaccine virus to a vaccine based on A/Perth/16/2009. The WHO announced this decision on 25 September 2009.

Our retrospective analysis allows us to see how the evolution of H3N2 continued after the VCM decision. Data collected after the vaccine selection deadline show that clade 158N/189K dominated for the following H3N2 seasons in both hemispheres, but two smaller clades each with HA1:145S mutations emerged from within this larger clade (Figure 3A). Although later HI measurements show that A/Perth/16/2009 did not cover later viruses from 158N/189K as effectively as it had covered earlier viruses from that clade (1.01 ± 1.22 log_2_ units), the new vaccine was still a better antigenic match than the previous two vaccines (Figure 3C). A/Perth/16/2009 did not effectively cover most viruses from the larger of the two derived clades, 145S.2 (1.94 ± 0.71 log_2_ units), indicating that the putative antigenic mutation defining the new clade might be responsible for antigenic drift. Despite this antigenic drift in a derived clade, A/Perth/16/2009 remained the WHO’s recommended H3N2 vaccine virus for the 2010–12 southern hemisphere seasons and the 2010-11 and 2011-12 northern hemisphere seasons (WHO, 2022).

### 3.3 Case study 2: Identification of genotype-specific patterns through visualization of raw data

Influenza researchers often define clades of H3N2 viruses based on the presence of mutations that have been previously shown to enable viruses to escape existing immunity (Wolf et al., 2006; Shih et al., 2007; Koel et al., 2013). The genetic similarity of viruses in the same clade typically corresponds with antigenic similarity of the same viruses as measured by HI assays. However, new mutations may arise within a clade that cause test viruses with those mutations to differ antigenically from earlier viruses in the same clade. Here, we demonstrate how aggregation of antigenic distances by clade can obscure the emergence of antigenically novel test viruses and how visualization of raw measurements can reveal these important patterns.

**FIGURE 4:**
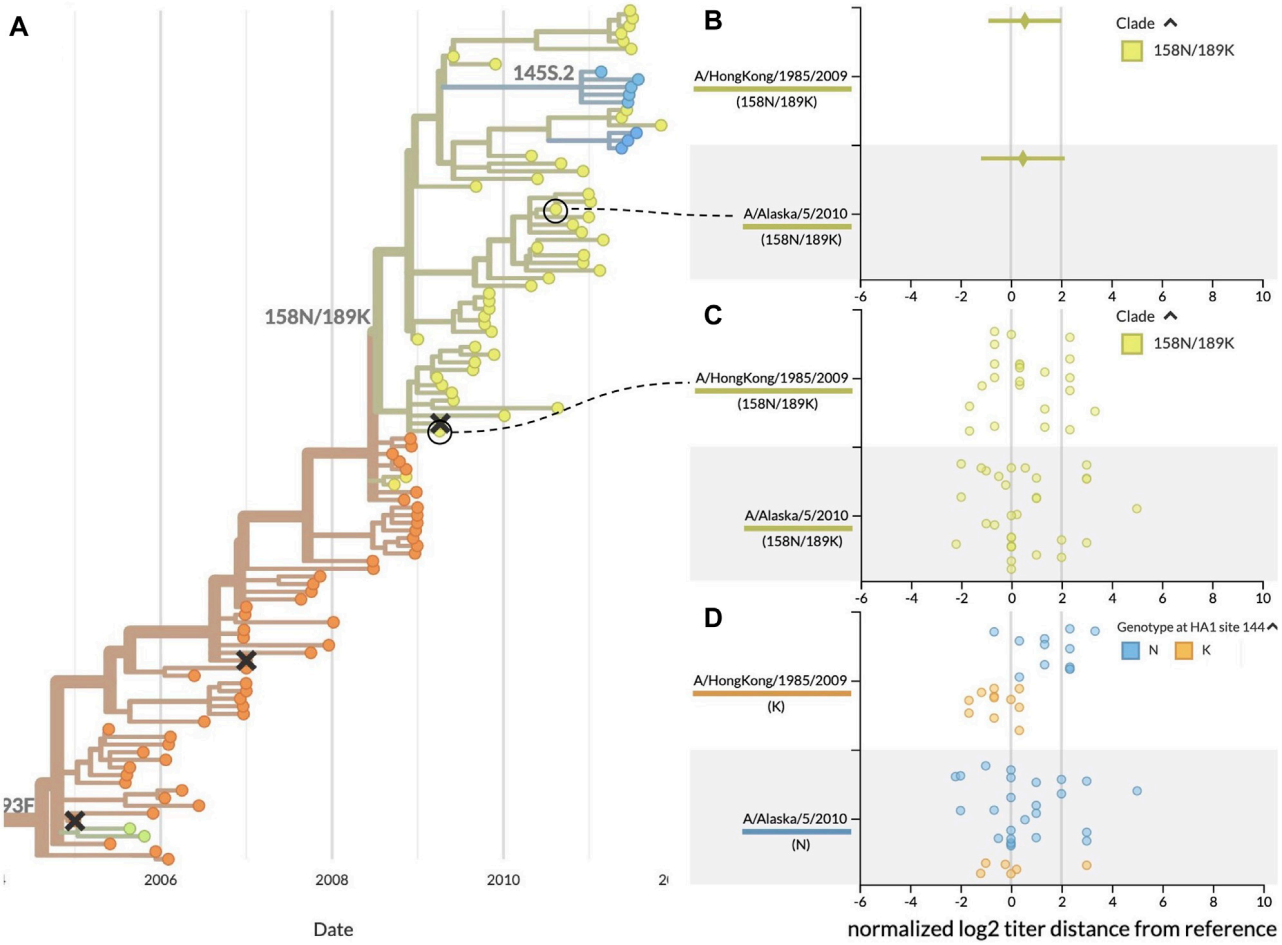
Antigenic distances from HI assays between clade 158N/189K reference and test viruses, highlighting two reference viruses with similar mean distances and raw distributions. **(A)** Summary phylogenetic tree with clade 158N/189K shown in yellowish green. **(B)** HI measurements when viewed as mean ± standard deviation show similar average values for two reference viruses and a wide range of antigenic diversity per reference. **(C)** Viewing the individual measurements reveals a previously hidden bimodal distribution in the measurements for both reference viruses. **(D)** Coloring individual measurements by genotypes at the putative antigenic site HA1:144 shows a potential genotype-specific explanation for the two clusters seen in the A/HongKong/1985/2009 measurements. In contrast, measurements for A/Alaska/5/2010 have a high variance that cannot be explained by the genotype of test viruses at HA1:144. This genotype-specific coloring reveals patterns that were not clear when coloring by clade alone.

Using the same data from the previous case study, we inspected the patterns of HI measurements for the clade at the highest global frequency during the fall 2009 vaccine composition meeting, 158N/189K. We identified a reference virus from each of the two largest subclades of 158N/189K where both viruses had similar average HI measurements and distributions of raw measurements (Figure 4B). Serum against A/HongKong/1985/2009 had a mean antigenic distance of 0.56 ± 1.45 log_2_ units to test viruses from 158N/189K, while serum against A/Alaska/5/2010 had a mean distance of 0.48 ± 1.66 log_2_ units. The distributions of raw HI measurements revealed clusters of values around titer distances of 0 and slightly above 2 for both reference viruses (Figure 4C). We hypothesized that these clusters could be explained by the presence of antigenic mutations in each reference virus’s subclade. The two largest subclades of 158N/189K were defined by mutations at HA1 positions 62, 144, and 212. Each of these positions were previously identified as a putative antigenic site in HA where mutations could enable escape from existing immunity (Wolf et al., 2006). Additionally, position 144 is immediately adjacent to HA1:145, a position previously identified to contribute to novel antigenic clusters (Koel et al., 2013). Based on this genetic information, we colored the HI measurements by the genotypes of the test viruses at position 144. The genotype-specific coloring showed two different alleles at position 144 including the ancestral allele 144N and the derived allele 144K (Figure 4D). This view also revealed that test viruses with same 144K genotype as A/HongKong/1985/2009 had lower antigenic distances (mean of −0.56 ± 0.75 log_2_ units), while viruses with the ancestral 144N genotype had higher antigenic distances (1.58 ± 1.14 log_2_ units). Interestingly, measurements against A/Alaska/5/2010 showed similar distances for both test viruses with 144N (0.57 ± 1.72 log_2_ units) and those with 144K (0.13 ± 1.51 log_2_ units). We observed the same patterns when grouping measurements by genotypes at the other two subclade-defining positions of HA1:62 and 212.

These results demonstrate how summary statistics can obscure biologically relevant patterns in the raw data. They also show how the ability to interactively color data by different viral attributes like genotype can produce hypotheses to explain the patterns we see in the raw data. For example, the genotype-specific patterns for A/HongKong/1985/2009 indicate the need for additional experiments to verify the antigenic effect of mutations at positions HA1:62, 144, and 212. In contrast, the high, genotype-independent variance of measurements against A/Alaska/5/2010 suggest that this reference virus might not be a stable vaccine candidate. This interactive visualization tool enables decision makers to explore their data and generate new hypotheses in ways that previous tools did not.

## 4 Discussion

Updating the seasonal influenza vaccine composition is a complex process that requires the synthesis of genetic and serological data and the interpretation of these data by a panel of international experts. Effective visualizations facilitate both the synthesis and interpretation by presenting data in a biologically meaningful context. Our interactive visualization tool presents serological data with a phylogenetic context, enabling decision makers to directly compare the antigenic distances between vaccine candidates and investigate patterns in the raw data. This tool regularly informs our discussions of influenza evolution with our collaborators in GISRS.

The move beyond static presentations of analyses towards interactive applications such as this facilitates more widespread usage and analysis of biological data. Specifically, the ability to link static views of the data, such as those found in VCM reports, with URLs that allow an interactive view into the data as presented in the figure is an important bridge between researchers. We hope that the adoption and continued development of biologically-informed visualization tools like this will facilitate a better understanding of pathogen evolution.

The benefits of integrated and interactive visualization of genetic and experimental data extend beyond serological measurements for seasonal influenza. High-throughput experimental measurements of mutational effects and immune escape in both seasonal influenza and SARS-CoV-2 have required custom tools for visualization and interpretation of these high-dimensional data (Hilton et al., 2020; Aksamentov et al., 2021; Garrett et al., 2021; Greaney et al., 2022). The data visualization presented here is amenable to showing similar multi-dimensional data, for instance linking different models to their scores across leaves in the phylogenetic tree in much the same way we have linked reference viruses to their titer measurements. The data structure for the measurements panel is purposefully agnostic to the pathogen or data generation approach. As research continues on the emergence of human pathogens from natural reservoirs in other organisms (Leendertz et al., 2016; Olival et al., 2017) and high-dimensional experimental measurements of these pathogens accumulate (Soh et al., 2019; Starr et al., 2022), this flexible data structure and the resulting interactive visualizations could impact decision-making related to pandemic preparedness.

## Data Availability

Details about how to prepare and analyze the data in this study live on GitHub at https://github.com/blab/measurements-panel and the dataset therein may be visualized *via* https://nextstrain.org/community/blab/measurements-panel/flu/seasonal/h3n2/ha
